# Beyond transcription factors: roles of mRNA decay in regulating gene expression in plants

**DOI:** 10.12688/f1000research.16203.1

**Published:** 2018-12-17

**Authors:** Leslie E Sieburth, Jessica N Vincent

**Affiliations:** 1 School of Biological Sciences, University of Utah, Salt Lake City, UT, USA

**Keywords:** mRNA decay, decapping, VCS, SOV, DIS3L2, P-bodies, gene expression

## Abstract

Gene expression is typically quantified as RNA abundance, which is influenced by both synthesis (transcription) and decay. Cytoplasmic decay typically initiates by deadenylation, after which decay can occur through any of three cytoplasmic decay pathways. Recent advances reveal several mechanisms by which RNA decay is regulated to control RNA abundance. mRNA can be post-transcriptionally modified, either indirectly through secondary structure or through direct modifications to the transcript itself, sometimes resulting in subsequent changes in mRNA decay rates. mRNA abundances can also be modified by tapping into pathways normally used for RNA quality control. Regulated mRNA decay can also come about through post-translational modification of decapping complex subunits. Likewise, mRNAs can undergo changes in subcellular localization (for example, the deposition of specific mRNAs into processing bodies, or P-bodies, where stabilization and destabilization occur in a transcript- and context-dependent manner). Additionally, specialized functions of mRNA decay pathways were implicated in a genome-wide mRNA decay analysis in Arabidopsis. Advances made using plants are emphasized in this review, but relevant studies from other model systems that highlight RNA decay mechanisms that may also be conserved in plants are discussed.

## Introduction

This review examines cytoplasmic mRNA decay with a focus on how mRNA decay regulates transcript abundance. Typically, changes in RNA abundances are attributed to transcription; however, considerable evidence supports important contributions from mRNA decay and this review focuses on recent advances in this area. Failing to account for changes in mRNA abundance that arise from altered decay rates can compromise molecular strategies for improving agriculture and ignores interesting biological phenomena.

**Figure 1.**
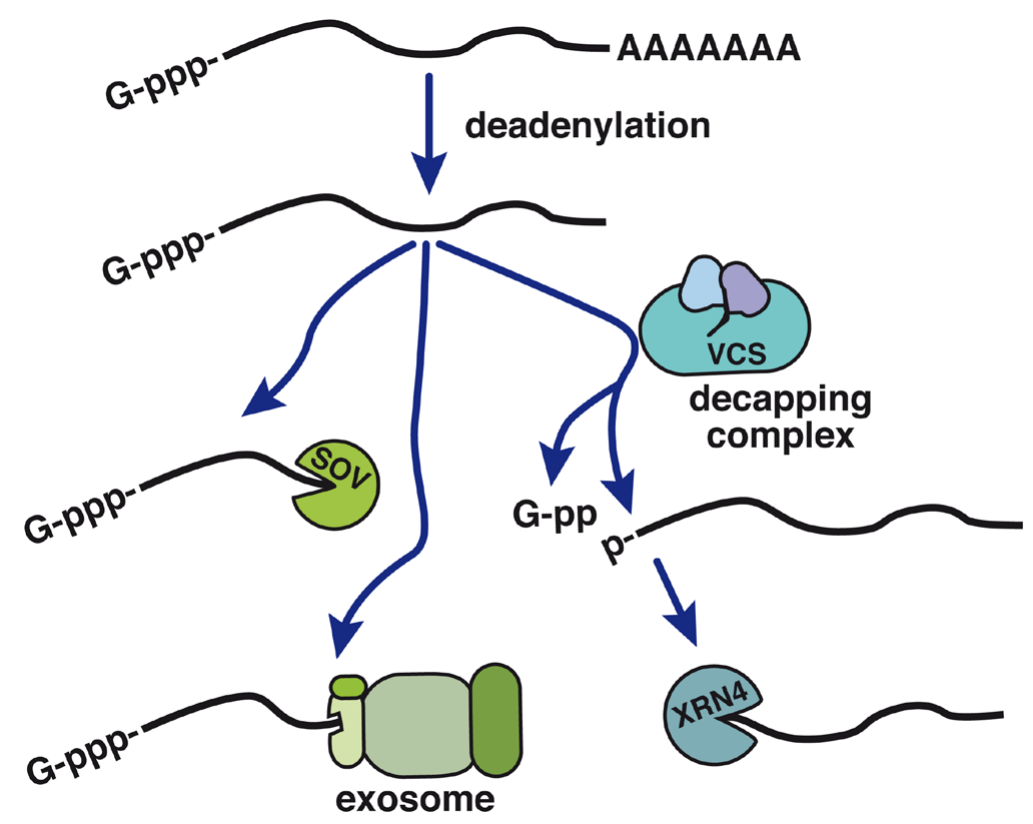
Schematic of the major cytoplasmic mRNA decay pathways. Mature mRNAs are first deadenylated by removal of the poly(A) tail. Deadenylated transcripts can be degraded in either the 3′→5′ or 5′→3′ direction. 3′→5′ degradation can occur by activity of the RNA exosome or by SUPPRESSOR OF VARICOSE (SOV). For degradation to occur in the 5′→3′ direction, transcripts must first be stripped of their 5′ m ^7^G cap by the RNA decapping complex, and further decay occurs by XRN4. VCS, VARICOSE.

Two stability determinants protect mRNA from untimely degradation: (1) the 3′ polyadenosine (poly(A)) tail and (2) the 5′ 7-methylguanosine (m ^7^G) cap. mRNA decay is initiated by the removal of the 3′ poly(A) tail in a process called deadenylation ^1^ ( Figure 1). Further degradation can act at the newly deadenylated 3′ end through the activity of the RNA exosome, which has distinct nuclear and cytoplasmic RNA decay and processing functions ^2^. Alternatively, 3′→5′ decay can occur via SUPPRESSOR OF VARICOSE (SOV), which is also known as DIS3-like 3′-5′ exoribonuclease 2 (DIS3L2) in fungi and metazoans. To initiate 5′→3′ decay, the m ^7^G cap is removed by the decapping complex, resulting in a 5′ monophosphorylated mRNA that is vulnerable to digestion by the cytoplasmic eXoRiboNuclease 4 (XRN4; XRN1 in fungi and metazoans). All three of these RNA decay pathways are highly conserved in eukaryotic model systems, with the exception of *Saccharomyces cerevisiae*, which lacks homologs of the decapping complex scaffold, VARICOSE (VCS), and SOV/DIS3L2. Thus, advances in any model system are of potential importance to the field.

### Deadenylation

Removal of the poly(A) tail of mRNA by deadenylases is the first and rate-limiting step in mRNA degradation ^1^. In plants, deadenylases include members of the CCR4-NOT complex, poly(A)-specific ribonuclease (PARN), and the poly(A) nuclease (PAN). CAF1 (of the CCr4-NOT complex) is a major deadenylase in plants, as loss-of-function mutants in this protein result in severely impaired mRNA decay ^3^. However, despite their importance for mRNA metabolism, the specificity of these three modes of deadenylation and their regulation are not well understood.

### 3′ to 5′ degradation

The RNA exosome is a large multi-subunit complex with both nuclear and cytoplasmic functions ^2^. Eukaryotic exosomes resemble the bacterial RNase PH and polynucleotide phosphorylase (PNPase) RNA decay complexes in that that they are large protein complexes with a barrel-like configuration ^4^. However, for bacterial PNPase, the barrel’s interior is the site of active RNA decay. This is in contrast to metazoan and fungal exosome core proteins, which lack catalytic activity, even though their sequences show some conservation ^5, 6^. In addition to the catalytically inactive central core (known as Exo9 for its nine subunits), the eukaryotic exosome contains peripheral subunits that carry out RNA processing and decay (for example, response regulator proteins 6 and 44 [Rrp6 and Rrp44]). A recent study showed that the Arabidopsis RRP41, an Exo9 subunit of the central barrel, retains catalytic activity ^7, 8^. This activity appears to extend through the entire plant lineage.

The other cytoplasmic 3′→5′ exonuclease, SOV/DIS3L2, is an exosome-independent enzyme that was first identified in Arabidopsis as an accession-specific suppressor of *vcs* mutants ^9^. It is a broadly conserved RNase II domain protein with highly conserved homologs in metazoans and fungi ^10– 13^. In these systems, SOV/DIS3L2 substrates include non-coding RNAs (ncRNAs), long non-coding RNAs (lncRNAs), microRNAs (miRNAs) and their precursors, and mRNAs ^14– 17^. *Drosophila sov/dis3l2* knock-down lines show over-growth phenotypes ^18^, and mutations in the *SOV/DIS3L2* gene in humans result in an embryonic-lethal cellular over-growth condition known as Perlman syndrome ^19, 20^. These severe phenotypes are in contrast to Arabidopsis, as several phenotypically normal accessions lack a functional SOV, including the Col-0 reference strain ^10^.

### 5′ to 3′ degradation

Removal of the 5′ m ^7^G cap of mRNAs is catalysed by DECAPPING2 (DCP2), which forms a heterodimer with its activator, DECAPPING1 (DCP1). In *S. cerevisiae*, dimerization of these two proteins is sufficient for decapping ^21^, but higher eukaryotes also require a scaffold protein, known as VCS (also referred to as human enhancer of decapping large subunit [HEDLS] or Ge-1 in other systems) ^9, 22– 25^. A notable feature of the mRNA decapping complex is that it can localize to cytoplasmic foci called processing bodies (P-bodies) ^9, 26^.

Decapped mRNAs are further degraded by XRN proteins, which also function in nuclear RNA metabolism (for example, RNA silencing, rRNA maturation, and transcription termination). XRN1 and XRN2 are the major 5′→3′ exonucleases in the fungal and metazoan cytoplasm and nucleus, respectively ^27^. Although plants do not possess an XRN1 ortholog, they do have an ortholog of XRN2, which is known as XRN4 and localizes to the cytoplasm and functions like XRN1 in other model systems ^28^.

The focus of this review is on regulation, a term we use in its strict sense: a tunable parameter that alters decay rates of specific mRNAs and results in changes in their abundance ^29^. Although endonucleolytic cleavage, which can be specified by small RNAs (including miRNAs), is an important regulatory process, space limitations prevent its inclusion here and so we refer readers to several excellent reviews ^30– 33^. We consider five emerging areas that reveal either regulation by decay or its potential: (1) RNA structure (covalent modifications and secondary structure), (2) RNA quality control (RQC), (3) regulation of mRNA decapping (including post-translational modifications of the decapping complex), (4) mRNA localization (to P-bodies, specifically), and (5) decay pathway interplay and the potential for an RNA to switch decay pathways.

## 1. RNA structure: covalent modifications and folding

Many RNA fates are determined by the activity of their binding proteins; different sets of proteins bind to RNAs as they progress from nascent transcripts through their eventual degradation. These proteins promote functions such as post-transcriptional processing, translation initiation, and targeting for decay. Recent progress in understanding the roles of covalent RNA modifications and their subsequent effects on the affinities of RNA-binding proteins and RNA secondary structure highlights their importance in regulating mRNA stability.

### N6-methyladenosine modification of mRNA

N6-methyladenosine (m ^6^A) is the most prevalent reversible covalent mark on eukaryotic RNA and plays important roles in many steps of RNA metabolism, including mRNA decay ^34^. Dynamic m ^6^A modification has been demonstrated to be vital for development, most notably cell differentiation ^35– 38^. Enzymes that catalyse the addition and removal of this modification (known as writers and erasers, respectively) have been characterized. Regulatory outcomes arise through reader proteins, which consist of YT521-B homology (YTH) domain proteins that bind m ^6^A-modified RNAs.

m ^6^A modification of eukaryotic mRNA occurs adjacent to stop codons and transcription start sites and within 3′ untranslated regions (UTRs) ^39^. Transcriptome-wide studies of m ^6^A modifications in Arabidopsis suggest that plants additionally have m ^6^A sites adjacent to start codons ^37, 40^. Accordingly, there are plant-specific modification motifs (such as URUAY) that are methylated along with the general eukaryotic RRACH and RAC consensus sequences ^41^. In fungi and metazoans, the YTHDF (reader) proteins remove the bound m ^6^A-modified transcripts from the translational pool and initiate their decay by recruiting the CCR4-NOT deadenylase complex ^42, 43^. A similar modification—2′-O-dimethyladenosine (m ^6^Am)—at, or adjacent to, the 5′ cap also impacts decay rates by inhibiting the action of the decapping enzyme (DCP2), thereby slowing decay rates ^44^.

EVOLUTIONARILY CONSERVED C-TERMINAL REGION 2 and 3 (ECT2 and ECT3) are cytoplasmic m ^6^A readers in plants that share homology with human YTHDF proteins ^45, 46^. Roles for plant m ^6^A readers have been implicated by developmental defects in mutants: plants mutant for the m ^6^A readers ECT2 and ECT3 have defects in leaf and trichome development ^41, 45, 46^. Plants mutant for components of the m ^6^A methyltransferase (writer) complex have hypomethylated transcripts and display enlarged shoot apical meristems and organogenesis defects due to increased stability of their target transcripts ^37, 38^. Mutants with defects in the m ^6^A eraser *ALKB HOMOLOG 10B* ( *ALKBH10B*) have suppressed vegetative growth and a delayed transition to flowering because of global hypermethylation ^47^, which was associated with stabilization of transcripts encoding FLOWERING LOCUS T (FT) and SQUAMOSA PROMOTER BINDING PROTEIN-LIKE 3 and 9 (SPL3/9). Thus, contrary to animals, m ^6^A modification in plants appears to stabilize target transcripts. This is emphasized by the observation that faster RNA decay also occurred in *ect2* reader mutants, indicating that the binding of ECT2 to m ^6^A-modified RNAs generally led to their stabilization ^40, 41^. Whether this pattern of stabilization by m ^6^A modification extends to all modified plant RNAs, and the mechanisms that bring about the differing responses in plants and other systems, will be an important topic of future exploration.

Roles of m ^6^A modification in many additional cellular responses, including viral responses in both plants and metazoans, have also been reported ^48, 49^. Teasing apart how cells integrate these reversible modifications and distinguish between selectively altered mRNA stability and other m ^6^A functions is likely to become a very interesting story.

### Uridylation

RNAs are also modified on their 3′ ends, and poly(A) tail addition is the best-known example. Recent studies highlight the importance of another 3′ modification, uridylation, which is catalysed by TERMINAL URIDYLYLTRANSFERASES (TUTases). UTP:RNA URIDYLTRANSERASE (URT1) and HEN1 SUPPRESSOR 1 (HESO1) are the two major TUTases in Arabidopsis ^50– 52^. Both URT1 and HESO1 uridylate miRNAs. In metazoan and fungal systems, miRNA uridylation leads to destabilization via SOV/DIS3L2 ^11, 12, 53^ but whether this is also the case in Arabidopsis is unknown.

mRNAs are also uridylated, which in fungal and metazoan systems is associated with destabilization. In Arabidopsis, URT1 is the major mRNA TUTase. It prevents trimming of the poly(A) tail and is also necessary to repair deadenylated RNAs ^54^. In other systems, mRNA uridylation has been linked to degradation by SOV/DIS3L2, including formation of SOV/DIS3L2-TUTase complexes ^16, 55^. However, whether mRNA uridylation in plants also leads to transcript destabilization is not known, perhaps because most Arabidopsis uridylation studies have used the Col-0 accession, which is an *sov* mutant ^10^. Finally, uridylation also tags the 5′ cleavage fragments of mRNAs that result from miRNA-induced cleavage. This feature promotes decay via RISC-INTERACTING CLEARING 3′-5′ EXORIBONUCLEASES1 and 2 (RICE1, 2) ^56^, which not only targets these fragments for decay but also appears to be important for allowing fast cycling of RISC complexes. In addition, non-stop decay (discussed in section 2, below) can eliminate 5′ cleavage products if miRNA or small interfering RNA (siRNA)-induced cleavage occurs in mRNA coding regions ^57^.

### RNA secondary structure

The complex folded structures of RNAs can also have important implications for stability. Analyses of RNA secondary structure in Arabidopsis found patterns of structure distributions in mRNAs, including less structure in the UTRs than in coding regions, and the observation that more structure generally resulted in lower transcript stability ^58– 60^. The impact of RNA structure and protein binding on RNA decay was recently explored in the context of root epidermal development. RNA secondary structures that were specific for root-hair or non-root-hair fates were found, and proteins that bound to these cell type–specific folds were identified ^61^. Interestingly, one of these interactions was with SERRATE, a zinc finger domain protein with functions in miRNA biogenesis, splicing, and epigenetic silencing ^62^, and appears to contribute to root-hair fate selection by miRNA-independent stabilization of specific mRNAs. Folded structures can also be impacted by m ^6^A modification, which can act as a structural switch by disrupting local secondary structures to promote interaction with RNA-binding proteins. These m ^6^A switches are enriched in 3′ UTRs and near stop codons, and switches located in introns were shown to play a role in alternative splicing ^39^. The refinement of methods for determining *in vivo* RNA structure promises many more insights ahead ^60, 63^.

## 2. RNA quality control

mRNAs that potentially encode aberrant protein products, such as truncated proteins caused by premature termination codons (PTCs) or by physical impediments to translation, or that disrupt ribosome homeostasis (due to lack of a start codon) are potentially deleterious for normal cellular function. Cells avoid these problems by identifying and degrading the offending mRNAs in a series of reactions known generally as RQC. These pathways include nonsense-mediated decay (NMD), which degrades RNAs with an abnormally positioned PTC; non-stop decay, which degrades mRNAs that lack a stop codon; and no-go decay, which degrades mRNAs with stalled ribosomes.

These pathways, however, have the potential to go well beyond a protective function and can contribute to regulation of mRNA abundance ^64^. For example, alternative splicing can lead to mRNA isoforms, including ones with PTCs. In plants, environmental stresses lead to enhanced production of mRNA variants containing PTCs, and their subsequent degradation allows adaptive responses to the initial stress ^65– 67^. Similarly, NMD selectively regulates transcript abundance of isoforms arising from alternative transcription start sites ^68^ and can also function in transcript autoregulation ^69^.

Another mechanism of RQC is the production of siRNAs that direct ARGONAUT-induced cleavage and decay of corresponding mRNAs ^70^. The accumulation of siRNAs has been observed in Arabidopsis mRNA decapping mutants and has been shown to elicit the severe phenotypes of decapping mutants because mutations in RNA-DEPENDENT RNA POLYMERASE 6 ( *rdr6*), which is required for siRNA amplification, suppress this severe phenotype ^71^. This result highlights the importance of mRNA decapping in modulating RNA abundances. Similarly, siRNA accumulates in double mutants that lack both XRN4 and SKI2 (a component of the RNA exosome), and the resulting severe phenotype was similarly alleviated by loss of RDR6 function ^72^. Thus, in addition to potential specialized functions, the major cytoplasmic mRNA decay pathways prevent generation of siRNAs.

## 3. Regulation of mRNA decapping

### Reversible phosphorylation

Each of the subunits of the mRNA decapping complex are subject to phosphorylation and these post-translational modification events have been implicated in regulating mRNA abundance ^73, 74^. DCP1 phosphorylation by MITOGEN-ACTIVATED PROTEIN KINASE 6 (MPK6) was shown to arise in response to dehydration. This modification is thought to slow mRNA decay, as transgenic lines with a non-phosphorylatable version of DCP1 showed slower decay of *EXPL1* RNA. Furthermore, rapid phosphorylations of VCS and DCP2 were found in a phosphoproteomic analysis of rapid responses to osmotic stress ^74^. The major site of VCS phosphorylation is in its S-rich linker domain. Because DCP2 requires DCP1 for activation and VCS serves as their interaction scaffold, phosphorylation of the S-rich linker could alter DCP2 activation. Salt stress similarly leads to VCS phosphorylation but via the SNF1-RELATED KINASE, SnRK2G ^75^. VCS and SnSRKG2 show constitutive physical interaction, and salt stress led the SnSRK2G-VCS complex to relocate to P-bodies ^75^. VCS phosphorylation was also correlated with changes in RNA decay rates, as a set of VCS-dependent RNAs decayed faster in wild-type (WT) (Col-0) plants following exposure to salt stress. This correlation suggests that VCS phosphorylation led to faster decay of these RNAs. Important questions for the future include determining how phosphorylation of the mRNA decapping complex subunits causes changes in mRNA decay; for example, is substrate recruitment affected or are mRNA decapping kinetics altered?

### Decapping activators

The SM-LIKE (LSM) complex consists of seven RNA-binding subunits and produces distinct nuclear and cytoplasmic complexes ^76– 78^. The cytoplasmic complex binds to the 3′ termini of oligoadenylated and deadenylated mRNA and recruits the mRNA decapping complex. Genetic analysis of the LSM complex of Arabidopsis revealed that this complex functions in decay and is necessary for normal responses to abiotic stresses, including high salinity and cold temperatures, because it regulates the targeting of mRNAs to the decapping complex ^79, 80^. A functionally related protein, protein associated with topoisomerase II 1 (PAT1), also has diverse functions in post-transcriptional regulation of RNA, including decay ^81^. In Arabidopsis, PAT1 has been implicated in pathogen response–based changes in gene expression. It is phosphorylated by MPK4, which causes its localization to P-bodies in response to challenge by bacteria and where it activates decay of specific mRNAs ^82^.

## 4. P-body localization

P-bodies form through phase separation of intrinsically disordered regions of proteins, such as the decapping complex subunits, and RNA ^83, 84^. These concentrated collections of decay enzymes and mRNAs have been generally considered to expedite RNA decay. However, in yeast and *drosophila*, mRNA decapping does not require formation of these bodies ^85, 86^. Recent studies using Arabidopsis have advanced our understanding of how specific mRNAs come to be localized in P-bodies. One participating protein is SPIRRIG (SPI), a BEACH-domain protein that interacts with DCP1 ^87^. SPI is required for localization of specific salt-response RNAs to P-bodies and for their stabilization. This compelling story is complicated by the multi-faceted functions of SPI, which also localizes to endosomes and is required for normal endosomal transport and vacuole morphology ^88^. The LSM proteins also have roles in localizing RNAs to P-bodies. These proteins interact to form heptameric rings, activate decapping, and associate with both mRNAs and the decapping complex in yeast ^89^. In Arabidopsis, the LSM proteins were shown to associate with stress-specific mRNAs and drive their localization into P-bodies, leading to faster transcript decay ^80, 90^. Although both SPI and LSM complexes move RNAs to P-bodies, their P-body localization has opposing effects on RNA stability. This raises many questions, including how P-bodies can be both stabilizing and destabilizing in an RNA-specific manner.

P-body studies using non-plant systems might offer some insight into these questions. A purification method was recently developed that allowed both proteomic and transcriptomic analyses of P-bodies isolated from human epithelial cells ^86, 91, 92^. This analysis confirmed P-body localization of mRNA decapping proteins, but there was no evidence that the localized mRNAs were undergoing decay. Instead, the results implicated P-bodies as an important site for translational arrest. However, whether P-bodies function similarly in all tissue types, and the extent to which human P-bodies can serve as a model for plants, needs to be determined. The stress-inducible P-bodies of yeast might be a more relevant model, even though *S. cerevisiae* lacks a homolog of the decapping complex scaffold, VCS. P-bodies of yeast have been shown to be sites of both decay and sequestration, and sequestered mRNAs can be restored to the translational pool ^93^. Understanding P-body functions and sorting out how P-body localization can lead to different RNA fates are important directions for future research.

## 5. Pathway interplay as a mechanism of selective regulation of mRNA decay

An under-explored aspect of mRNA decay is whether the three major cytoplasmic decay pathways ( Figure 1) have unique functional or regulatory significance. To identify their substrates, our lab carried out a genome-wide mRNA decay analysis using four Arabidopsis genotypes: a synthetic WT (Col-0 carrying a functional L. *er SOV* transgene), *vcs* and *sov* single mutants, and a *vcs sov* double mutant ^94^. Contributions of decapping (VCS) and SOV to the decay of mRNAs followed the assumption that mRNA substrates of decapping (VCS) and SOV would decay more slowly in *vcs* and *sov* mutants, respectively. We found that most RNAs decay by combined contributions of two or more pathways. While decapping (VCS) is required to sustain normal decay for 67% of the 17,293 analyzed RNAs, few were solely dependent on decapping for their decay. In addition, VCS-dependent RNAs tend to decay quickly, have abundances that are responsive to stress or developmental signals, and/or encode transcription factors ^94^. Decay of 22% of the analyzed transcripts was not attributable to either VCS (decapping) or SOV, suggesting a large role for the RNA exosome. In contrast to decapping (VCS)-dependent RNAs, putative exosome substrates were generally slow-decaying RNAs that encode proteins with housekeeping functions. Thus, both mRNA decapping and the RNA exosome are specialized in terms of mRNA substrate functions and decay rates.

**Figure 2.**
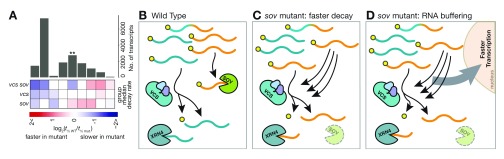
Loss of SUPPRESSOR OF VARICOSE (SOV) induces RNA decay feedback in *Arabidopsis*. ( **A**) Heat map depicts RNA decay rates, relative to the wild type, and histogram indicates the degree to which each pattern was represented. Bar with two asterisks indicates RNAs with VARICOSE (VCS)-dependent faster decay rates in *sov* mutants. ( **B**) Diagram of VCS and SOV decay in wild type. Yellow circles represent the 5′ m ^7^G cap, blue RNAs decay by mRNA decapping, orange RNAs by SOV, and blue-orange gradient colored RNAs are substrates of both pathways. ( **C**) In *sov* mutants, some RNAs that are normally substrates of SOV instead decay by mRNA decapping, and they decay faster. ( **D**) In *sov* mutants, faster-decay RNAs maintain a normal abundance, indicating transcriptional feedback, which is also called RNA buffering.

The search for mRNA substrates that decayed more slowly in *sov* mutants initially suggested that SOV/DIS3 contributes to decay of only about 9% of the analyzed transcripts ^94^. Curiously, 33% of these RNAs decay much faster in *sov* mutants than in WT. This faster decay comes from compensatory activity of the mRNA decapping complex, as indicated by slower decay of these same transcripts in *vcs sov* double mutants ( Figure 2A). Because many of the affected RNAs are not normally substrates of decapping, these findings suggest that transcripts that are normally substrates of SOV can become decapping substrates in its absence. Furthermore, the observation that mRNA decapping was associated with fast-decaying RNAs was supported because after these RNAs switched to the decapping pathway, their decay rates were much faster. We interpret this compensation by an alternate decay pathway as the activation of a feedback mechanism that compensates for the loss of SOV ( Figure 2B, C). Thus, triggering feedback results in a subset of the SOV substrates switching to decapping-mediated decay. This interpretation implicates that SOV actually contributes to decay of about 42% of the analyzed transcripts. Among the many questions raised by this analysis are whether the plasticity of mRNA decay pathways shown by SOV substrates extends more broadly across the transcriptome and whether pathway plasticity is used to regulate mRNA decay rates.

The *sov*-triggered feedback also appears to result in mRNA buffering, as indicated by near WT abundances despite much faster decay ^94^. This requires a commensurate increase in transcription and thus communication from cytoplasmic decay to the transcriptional machinery ( Figure 2D). A similar feedback pathway that coordinates transcription and decay has been described in yeast ^95, 96^. This RNA buffering system appears to explain why some Arabidopsis accessions tolerate mutations in *SOV/DIS3L2*.

## Outlook

RNA decay pathways are highly conserved across eukaryotes, and research using Arabidopsis continues to contribute strongly to this field, as the genetic resources for studying mRNA decay in Arabidopsis make it an outstanding choice. However, many mRNA decay studies appear to be technically and computationally challenged. Selection of time points can have enormous implications on outcomes and accordingly should be selected on the basis of mRNA half-life range. Similarly, quantitative approaches to data analysis can compromise data outcomes. Pools of RNA change over time and can lead to the false impression that the abundance of stable RNAs increase. Furthermore, slight differences in measured decay rates can be difficult to assess. We have generated an R package in Bioconductor (RNAdecay) that, among other things, assists with normalization and allows statistical comparisons between treatments. This resource is freely available through Bioconductor ( https://bioconductor.org/packages/release/bioc/html/RNAdecay.html).

Recent discoveries have led to the identification of novel regulatory mechanisms for mRNA decay, including uridylation, methylation, and the potential for mRNAs to switch between decay pathways. Similarly, recent discoveries have led to the reconsideration of some past concepts, including P-bodies and the functional consequences of localized mRNAs. However, most studies address the behavior of only a few mRNAs, cell types, or a single condition, limiting the generality of outcomes. As costs for genome-wide approaches continue to decline, we can look forward to a clearer picture of the flexibility of mRNA stability, mechanisms of stability control, and positioning decay in the overall control of mRNA abundance.
